# Activin A increases phagocytosis of *Escherichia coli* K1 by primary murine microglial cells activated by toll-like receptor agonists

**DOI:** 10.1186/s12974-018-1209-2

**Published:** 2018-06-07

**Authors:** Catharina Diesselberg, Sandra Ribes, Jana Seele, Annika Kaufmann, Sandra Redlich, Stephanie Bunkowski, Uwe-Karsten Hanisch, Uwe Michel, Roland Nau, Sandra Schütze

**Affiliations:** 1 Institute of Neuropathology, University Medical Center Göttingen, Robert-Koch-Str. 40, 37075 Göttingen, Germany; 2 Department of Geriatrics, Evangelisches Krankenhaus Göttingen-Weende, An der Lutter 24, 37075 Göttingen, Germany; 3 Department of Neurology, University Medical Center Göttingen, Robert-Koch-Str. 40, 37075 Göttingen, Germany; 4 Department of Geriatrics, AGAPLESION Frankfurter Diakonie Kliniken, Wilhelm-Epstein-Str. 4, 60431 Frankfurt am Main, Germany

**Keywords:** Bacterial meningitis, Activin, Proinflammatory cytokines, TLR, Innate immune system, Nitric oxide, CNS infection, Phagocytosis, *E. coli*

## Abstract

**Background:**

Bacterial meningitis is associated with high mortality and long-term neurological sequelae. Increasing the phagocytic activity of microglia could improve the resistance of the CNS against infections. We studied the influence of activin A, a member of the TGF-β family with known immunoregulatory and neuroprotective effects, on the functions of microglial cells in vitro.

**Methods:**

Primary murine microglial cells were treated with activin A (0.13 ng/ml–13 μg/ml) alone or in combination with agonists of TLR2, 4, and 9. Phagocytosis of *Escherichia coli* K1 as well as release of TNF-α, IL-6, CXCL1, and NO was assessed.

**Results:**

Activin A dose-dependently enhanced the phagocytosis of *Escherichia coli* K1 by microglial cells activated by agonists of TLR2, 4, and 9 without further increasing NO and proinflammatory cytokine release. Cell viability of microglial cells was not affected by activin A.

**Conclusions:**

Priming of microglial cells with activin A could increase the elimination of bacteria in bacterial CNS infections. This preventive strategy could improve the resistance of the brain to infections, particularly in elderly and immunocompromised patients.

## Background

Bacterial meningitis is associated with high mortality and long-term neurological sequelae despite the use of bactericidal antibiotics [1, 2]. The incidence and the frequency of an unfavorable outcome of bacterial central nervous system (CNS) infections are increased in immunocompromised and aged persons. In these patients, the Gram-negative bacterium *Escherichia coli* is one of the most prevalent causative pathogens [3, 4]. The presence of the polysaccharide capsule K1 allows *E. coli* strains to survive in the bloodstream, to cross the blood-brain barrier by penetrating the brain microvascular endothelial cell layer and to enter the CNS [5]. In the CNS, meningeal and perivascular macrophages and microglia, the resident immune cells and the major constituents of innate immunity in the brain parenchyma, represent the first line of defense against bacteria [6]. They express toll-like receptors (TLR) that recognize pathogen-associated molecular patterns (PAMPS) [7, 8]. TLR on microglia are stimulated during the early phase of CNS infections and systemic infections [9, 10]. TLR2 is activated by bacterial lipopeptides [11], TLR4 recognizes endotoxin (LPS) [12], and TLR9 is activated by bacterial DNA [13]. In response to an inflammatory stimulus, microglia undergo changes in morphology and functions, such as production of proinflammatory cytokines, chemokines and reactive oxygen species (ROS), phagocytic activity, antigen presentation, clearance of toxic cellular debris, and promotion of tissue repair [14–16].

We previously demonstrated that the age-related decline of microglia and macrophage functions, particularly the age-related decline of their phagocytic capacity, plays an essential role for the impaired elimination of bacteria and the higher mortality after an intracerebral bacterial challenge in aged mice [17]. Thus, strategies to increase the phagocytic potential of macrophages and microglial cells appear promising for the prevention and therapy of CNS infections, especially in elderly and immunocompromised patients. On the other hand, stimulation of microglial cells bears the risk of microglia-mediated neuronal damage.

In vitro, activation of microglial cells with agonists of TLR 2, 4, and 9 increases phagocytosis and intracellular killing of *E. coli* K1 [18]. However, microglia activated by these TLR agonists also produce proinflammatory cytokines (e.g., TNF-α, IL-6, CXCL1) and nitric oxide (NO) [18–20] which can cause destruction of neuronal axons and somata [21–25]. Palmitoylethanolamide (PEA) enhances phagocytosis of *E. coli* K1 by microglial cells in vitro without inducing the release of proinflammatory cytokines and increases survival in mouse models of *E. coli* meningitis and sepsis [26]. The identification of other compounds which increase phagocytosis of pathogens without exerting collateral damage to the brain tissue is a promising approach for the prophylaxis and early therapy of intracerebral infections in high-risk individuals [27].

We considered activin A an ideal compound for this purpose, as it has been closely linked to bacterial CNS infections and microglial cells, and both immunoregulatory and neuroprotective effects have been described (for review, see [28, 29]). Activin A is a multifunctional member of the TGF-β-superfamily [30]. Together with its binding protein follistatin, activin A is involved in the fine-tuning of the host’s inflammatory response [28, 31]. Levels of activin A and follistatin are elevated in serum during sepsis [32] and in CSF during meningitis [33, 34]. Depending on the circumstances, activin A can be both pro- or anti-inflammatory by regulating key mediators of the inflammatory response such as cytokines and [28, 31]. Microglial cells have been shown to be a source of activin A during bacterial infections [32, 35, 36], as well as a target of activin A. Microglia express activin A receptor type II (Act-RII) and Act-RI [36, 37] by which Smad and non-Smad signaling pathways are initiated. In several experiments with murine peritoneal macrophages and macrophage cell lines, activin A modulated not only the release of cytokines and ROS but also the phagocytic activity as assessed by the uptake of chicken red blood cells (cRBC) or latex particles. Results from these studies indicate that activin A increases the phagocytic capacity of resting macrophages [38–40] and inhibits the phagocytic activity of LPS-activated macrophages [40, 41]. To our knowledge, the effect of activin A on phagocytosis of a living bacterium and on the phagocytic activity of microglia has not been examined so far.

Here, we investigated the effect of activin A on resting and activated primary murine microglial cells with a focus on their ability to phagocytose *E. coli* K1.

## Methods

### Primary murine microglia cell cultures

Primary cultures of microglial cells were prepared from brains of newborn C57BL/6 mice (1–3 days) [19]. After removal of the meninges, cells were mechanically dissociated and suspended in Dulbecco’s modified Eagle’s medium (DMEM) with Glutamax I (Gibco, Karlsruhe, Germany) supplemented with 10% fetal calf serum (FCS), 100 U/ml penicillin, and 100 μg/ml streptomycin. Cells were plated at a density of two brains per T75 culture flask (Corning Costar GmbH, Wiesbaden, Germany) and incubated at 37 °C in a humid atmosphere with 5% CO_2_. Culture medium was changed twice a week. After 10–14 days, microglial cells were separated from the confluent astrocyte layer by shaking 200×/min for 30 min and plated in 96-well plates at a density of 50,000 cells/well for phagocytosis experiments. For staining, microglial cells were seeded on coverslips placed in a 24-well plate at a density of 50,000 cells/well.

### Stimulation of microglial cells

Two hours after plating, cells were treated with activin A (R&D Systems, Wiesbaden, Germany) for 24 h. Different concentrations of activin A were chosen for the experiments: 0.13 ng/ml (= 10 pM), 1.3 ng/ml (= 100 pM), 13 ng/ml (= 1 nM), and a high concentration of 13 μg/ml (= 1 μM). Unstimulated control cells were treated with medium only. After 24 h, microglial cells were additionally treated with tripalmitoyl-S-glycerl-cysteine (Pam_3_CSK_4_; EMC Microcollections, Tübingen, Germany) as an agonist of TLR1/2, endotoxin (lipopolysaccharide, LPS) from *E. coli* serotype 026:B6 (Sigma-Aldrich, Taufkirchen, Germany) as an agonist of TLR4, or CpG oligodesoxynucleotide (ODN) 1668 (TCC ATG A**CG** TTC CTG ATG CT) containing unmethylated cytosine-guanosine motifs (CpG; TIB Molbiol, Berlin, Germany) as an agonist of TLR9 for 24 h. Concentrations of the different TLR agonists which induced a 2- to 3-fold increase of phagocytosis of *E. coli* K1 in our previous experiments were used [18, 20]. Control cells were treated with medium only. Treatment with 1 μg/ml LPS was used as a positive control of stimulation based on previous experiments [18, 19]. In experiments for measurement of NO release, microglial cells were additionally stimulated with 100 U/ml interferon-γ, because a basal level of interferon-γ is essential to reach a substantial NO release of microglial cells after stimulation [15] Unstimulated cells were treated with medium containing 100 U/ml interferon-γ only. After 24 h of stimulation, supernatants were stored at − 80 °C until measurement of NO, cytokine, and chemokine levels. Cells were used for the bacterial phagocytosis assay, the cell viability assay, or were fixated in 4% formaldehyde for staining.

### Bacteria

The *E. coli* strain K1 (serotype O18:K1:H7) originally isolated from the cerebrospinal fluid of a child with neonatal meningitis (gift of Dr. Gregor Zysk, Institute for Medical Microbiology, Düsseldorf, Germany) was used for phagocytosis experiments. Bacteria were grown over night on blood agar plates, harvested in 0.9% saline, and stored at − 80 °C. Frozen aliquots were thawed directly before the experiment and diluted with saline to the required bacterial concentration.

### Phagocytosis assay

The phagocytosis assay was performed as previously described [18]. After stimulation, microglia were exposed to the encapsulated *E. coli* K1 for 90 min at 37 °C, 5% CO_2_ with a ratio of 100 bacteria per phagocyte (5 × 10^6^ colony-forming units (CFU)/well). After incubation with bacteria, microglial cells were washed with PBS and incubated with DMEM containing gentamicin (final concentration 100 μg/ml; Sigma-Aldrich, Taufkirchen, Germany) for 60 min to kill extracellular bacteria. Thereafter, cells were washed twice with PBS and lysed with 100 μl of distilled water. The number of intracellular bacteria was determined by quantitative plating of serial 1:10 dilutions of the lysate on blood agar plates.

### Cell viability test

Cell viability of microglial cells was determined using the WST-1 Cell Proliferation Reagent (Roche Applied Science, Mannheim, Germany). The assay is based on the cleavage of the tetrazolium salt WST-1 by active mitochondria producing a soluble formazan. This conversion only occurs in viable cells. Cells were incubated with WST-1 for 2 h. Then, the formazan dye formed was quantified by measuring the optical density at 490 nm using a Genios multiplate reader (Tecan, Crailsheim, Germany). The absorbance directly correlated with the number of metabolically active cells.

### Cytokine and chemokine measurements

Concentrations of tumor necrosis factor alpha (TNF-α), interleukin-6 (IL-6), and the chemokine (C-X-C motif) ligand 1 (CXCL1, also called GRO α or KC) in the cell culture supernatants were measured by ELISA. TNF-α levels were determined using antibody pairs from BioLegend (Biozol, Munich, Germany), and DuoSet ELISA Development Kits (R&D Systems, Wiesbaden, Germany) were used for the measurement of IL-6 and CXCL1. The color reaction was measured at 450 nm in a microplate reader (Bio-Rad, Munich, Germany). Detection limits were 19 pg/ml for TNF-α, 38 pg/ml for IL-6, and 64 pg/ml for CXCL1.

### Quantification of nitric oxide release

NO release was quantified by the measurement of nitrite, one of its stable reaction products, in the supernatant of microglial cultures using the Griess reagent. One hundred microliters of the supernatant were mixed with 100 μl Griess reagent [equal volumes of 1% sulfonilamide in 30% acetate and 0.1% *N*-(1-naphthyl) ethylenediamine in 60% acetate] in a 96-well plate. After 10 min, the optical density at 570 nm was measured with a Genios multiplate reader (Tecan, Crailsheim, Germany). Concentrations were calculated by comparison of absorptions with a standard curve.

### Isolectin B4 staining

Isolectin B4 staining was used to assess the purity and density of microglial cultures and the morphology of microglial cells. For this purpose, microglial cells were plated on poly-l-lysine-coated cover slips. After stimulation, cells were fixated in 4% formalin. Fixated cells were permeabilized with Triton X (0.1% in PBS) for 30 min and then incubated with biotinylated isolectin B4 (5 μg/ml, diluted in PBS + 1% BSA; Sigma-Aldrich, Taufkirchen, Germany) for 90 min. Thereafter, cells were treated with avidin-biotin complex (ABC, Vector, Burlingame, CA) for 30 min, and diaminobenzidine was used for visualization (5 min) resulting in a brown staining of the somata of microglial cells. The purity of microglia in the cultures was greater than 98%.

### Statistics

GraphPad Prism 5.0 Software (GraphPad Software, San Diego, CA, USA) was used to perform statistical analyses and graphical presentation. Parametric data were expressed as means ± standard deviations (SD); nonparametric data were expressed as medians (25th percentile/75th percentile). Student’s *t* test was performed to compare two groups of parametric data; ANOVA followed by Bonferroni’s multiple comparison test was used to compare more than two groups of parametric data. Nonparametric data were analyzed by Mann-Whitney *U* test or Kruskal-Wallis test followed by Dunn’s multiple comparison test. *P* values < 0.05 were considered statistically significant.

## Results

### Activin A alone did not influence phagocytosis of *E. coli* K1 by primary murine microglial cells

**Fig. 1:**
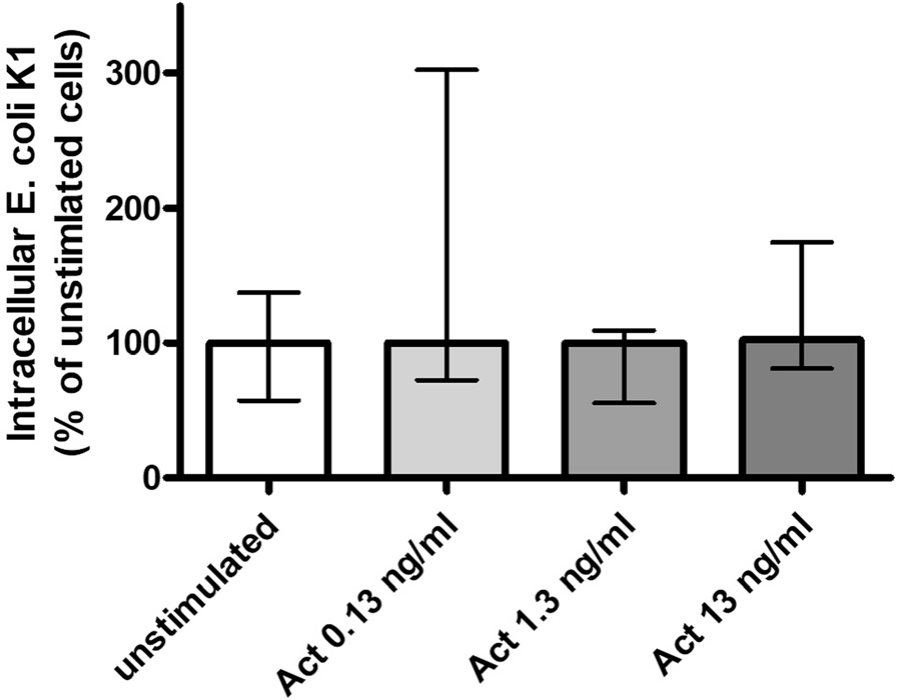
Phagocytosis of *E. coli* by microglial cells treated with activin A alone. Comparison of *E. coli* K1 phagocytosis between unstimulated microglial cells and microglial cells treated with activin A in concentrations of 0.13, 1.3, and 13 ng/ml (*n* = 11–30 from up to seven experiments). In each experiment, the mean number of bacteria ingested by unstimulated control cells was defined as 100%. Phagocytic rates of the stimulated groups are presented as percentages of phagocytosis of the unstimulated control group (medians with interquartile ranges). Data were analyzed by Kruskal-Wallis test followed by Dunn’s multiple comparison test

Treatment of microglial cells with activin A alone in concentrations between 0.13 and 13 ng/ml (Fig. 1) and in a high concentration of 13 μg/ml (Table 1) did not influence phagocytosis of *E. coli K1*.

**Table 1:** Phagocytosis of *E. coli* K1 and release of proinflammatory cytokines and NO by microglial cells treated with 13 μg/ml activin A alone and in combination with different TLR agonists

	No stim.	Act A 13 μg/ml	P3C	P3C + Act A	LPS	LPS + Act A	CpG	CpG + Act A
Phagocytosis (%)	100 (52/143)	56 (27/96)	337 (147/561)	276 (219/500)	493 (324/750)	275 (180/949)	493 (270/1399)	750 (183/7914)
	*p* = 0.11		*p* = 0.85		*p* = 0.33		*p* = 0.94	
TNF-α (pg/ml)	125 (85/195)	112 (33/157)	7280 (1884/8315)	4046 (1615/7189)	8103 (7126/8636)	4776 (1255/5505)	4325 (932/4829)	4826 (1313/7199)
	*p* = 0.34		*p* = 0.31		*p* < 0.0003***		*p* = 0.11	
IL-6 (pg/ml)	38 (38/95)	38 (38/38)	38 (38/7383)	2785 (38/5913)	3910 (2832/4192)	2017 (1894/2215)	656 (435/727)	751 (656/941)
	*p* = 0.56		*p* = 0.93		*p* = 0.10		*p* = 0.27	
CXCL1 (pg/ml)	928 (352/1536)	690 (64/1656)	9168 (1908/19508)	6445 (2051/14133)	2802 (2751/3003)	3204 (2412/4460)	6650 (4309/8876)	4410 (4108/8820)
	*p = 0.77*		*p = 0.82*		*p = 0.70*		*p = 0.70*	
NO (μM)	4.6 (4.0/5.3)	4.0 (3.7/4.9)	32.4 (22.0/34.7)	40.5 (29.4/42.8)	26.1 (18.7/28.2)	31.6 (28.0/32.8)	24.4 (15.0/26.5)	25.4 (23.8/32.2)
	*p* = 0.45		*p* = 0.03*		*p* = 0.02*		*p* = 0.11	

Phagocytosis of *E. coli* K1 by primary murine microglial cells [percentages of phagocytosis of the unstimulated control group (medians with interquartile ranges); *n* = 9–16 from up to four experiments] as well as concentrations (medians with interquartile ranges) of TNF-α (pg/ml), IL-6 (pg/ml), CXCL1(pg/ml), and NO (μM) in the supernatants after treatment with 13 μg/ml activin A (Act A) alone and in combination with the TLR2 agonist Pam_3_CSK_4_ (P3C), the TLR4 agonist LPS, and the TLR9 agonist CpG. Data were analyzed by Mann-Whitney *U* test (*p < 0.05, ***p < 0.001)

### Activin A dose-dependently enhanced phagocytosis of *E. coli* K1 by microglial cells stimulated with agonists of TLR2, 4 and 9

**Fig. 2:**
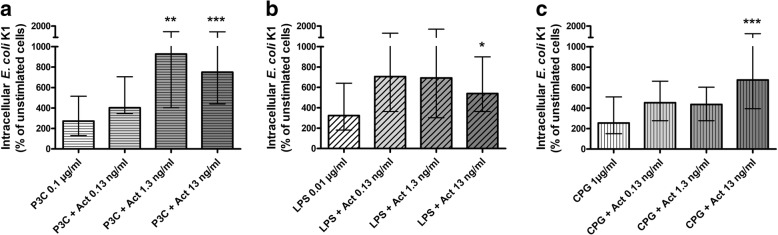
Phagocytosis of *E. coli* by microglial cells treated with activin A and different TLR agonists. Comparison of *E. coli* K1 phagocytosis between microglial cells treated with activin A in concentrations of 0.13, 1.3, and 13 ng/ml in combination with the TLR2 agonist Pam_3_CSK_4_ (P3C; 0.1 μg/ml, **a**), the TLR4 agonist LPS (0.01 μg/ml, **b**), and the TLR9 agonist CpG (1 μg/ml, **c**), and microglial cells treated with the respective TLR agonist alone (*n* = 14–30 from up to seven experiments). Data were analyzed by Kruskal-Wallis test followed by Dunn’s multiple comparison test (*p < 0.05; **p < 0.01, ***p < 0.001). In each experiment, the mean number of bacteria ingested by unstimulated control cells was defined as 100%. Phagocytic rates of the stimulated groups are presented as percentages of phagocytosis of an unstimulated control group (medians with interquartile ranges). Please mind the interrupted *y*-axis

Stimulation with the different TLR agonists increased phagocytosis of *E. coli K1* by microglial cells compared to unstimulated cells. Based on previous experiments [18], concentrations of TLR agonists evoking an approximately 3-fold increase of phagocytosis were chosen: Pam_3_CSK_4_ 0.1 μg/ml: 273 (129/515) %, LPS 0.01 μg/ml: 324 (182/641) %, CpG 1 μg/ml: 255 (150/510) % (Fig. 2a–c).

Additional treatment of microglial cells with activin A in concentrations of 1.3 and 13 ng/ml starting 24 h before stimulation with TLR agonists further increased phagocytosis of *E. coli* K1 compared to treatment with the respective TLR agonists alone (Fig. 2a–c). Compared to treatment with Pam_3_CSK_4_ alone [273 (129/515) %], additional treatment with activin A 1.3 ng/ml increased the phagocytic rate 3.4-fold [927 (403/1503) %, *p* < 0.01] and activin 13 ng/ml increased the phagocytic rate 2.8-fold [750 (440/1490) %, *p* < 0.001; Fig. 2a]. Compared to treatment with LPS alone [324 (182/641) %], additional treatment with activin 13 ng/ml increased the phagocytic rate 1.7-fold [540 (364/900) %, *p* < 0.05; Fig. 2b]. Compared to treatment with CpG alone [255 (150/510) %], additional treatment with activin 13 ng/ml increased the phagocytic rate 2.7-fold [676 (393/1337) %, *p* < 0.01; Fig. 2c].

Additional treatment of microglial cells with activin A in the high concentration of 13 μg/ml starting 24 h before stimulation with TLR agonists did not significantly influence phagocytosis of *E. coli K1* compared to treatment with the respective TLR agonists alone (Table 1).

### In concentrations enhancing phagocytosis, activin A did not increase the release of proinflammatory cytokines and nitric oxide by microglial cells

**Fig. 3:**
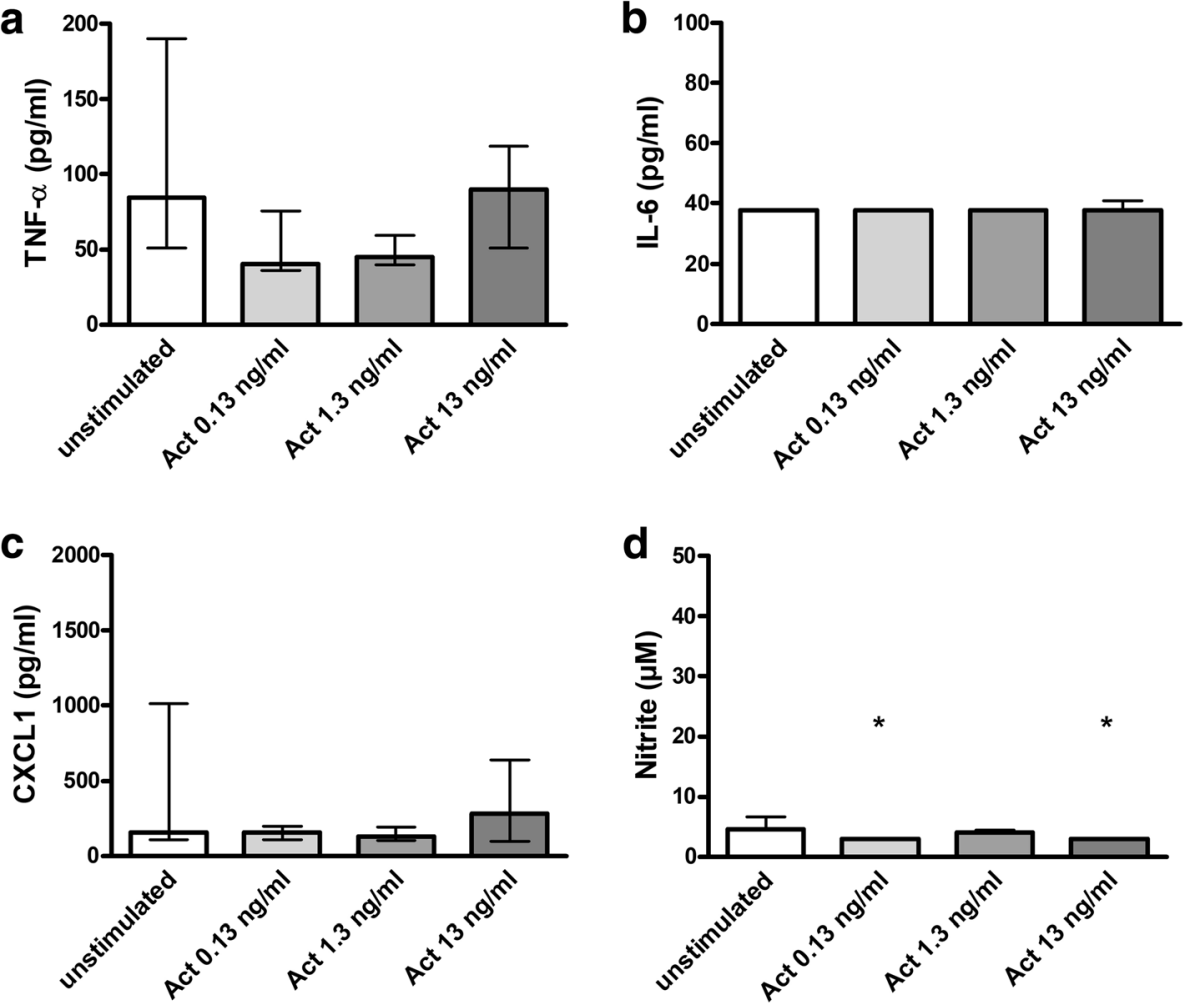
Release of proinflammatory cytokines and NO by microglial cells treated with activin A alone. Comparison of concentrations of TNF-α (pg/ml) (**a**), IL-6 (pg/ml) (**b**), CXCL1 (pg/ml) (**c**), and NO (μM) (**d**) in the supernatants of unstimulated primary murine microglial cells and microglial cells treated with activin A in concentrations of 0.13, 1.3, and 13 ng/ml [**a**: *n* = 8–27 from up to six experiments; **b**, **c**: *n* = 8–18 from up to four experiments; **d**: *n* = 3 from one single experiment / **b**: almost all values were below the detection limit of 38 pg/ml IL-6; **d**: for activin A 0.13 and 13 ng/ml, all values were below the detection limit of 3 μM nitrite]. Data are presented as medians with interquartile ranges and were analyzed by Kruskal-Wallis test followed by Dunn’s multiple comparison test (*p < 0.05)

Treatment of microglial cells with activin A alone in concentrations between 0.13 and 13 ng/ml did not significantly influence the release of TNF-α (Fig. 3a), IL-6 (Fig. 3b), and CXCL1 (Fig. 3c) and slightly decreased the release of NO (activin A 0.13 and 13 ng/ml: *p* < 0.05; Fig. 3d).

**Fig. 4:**
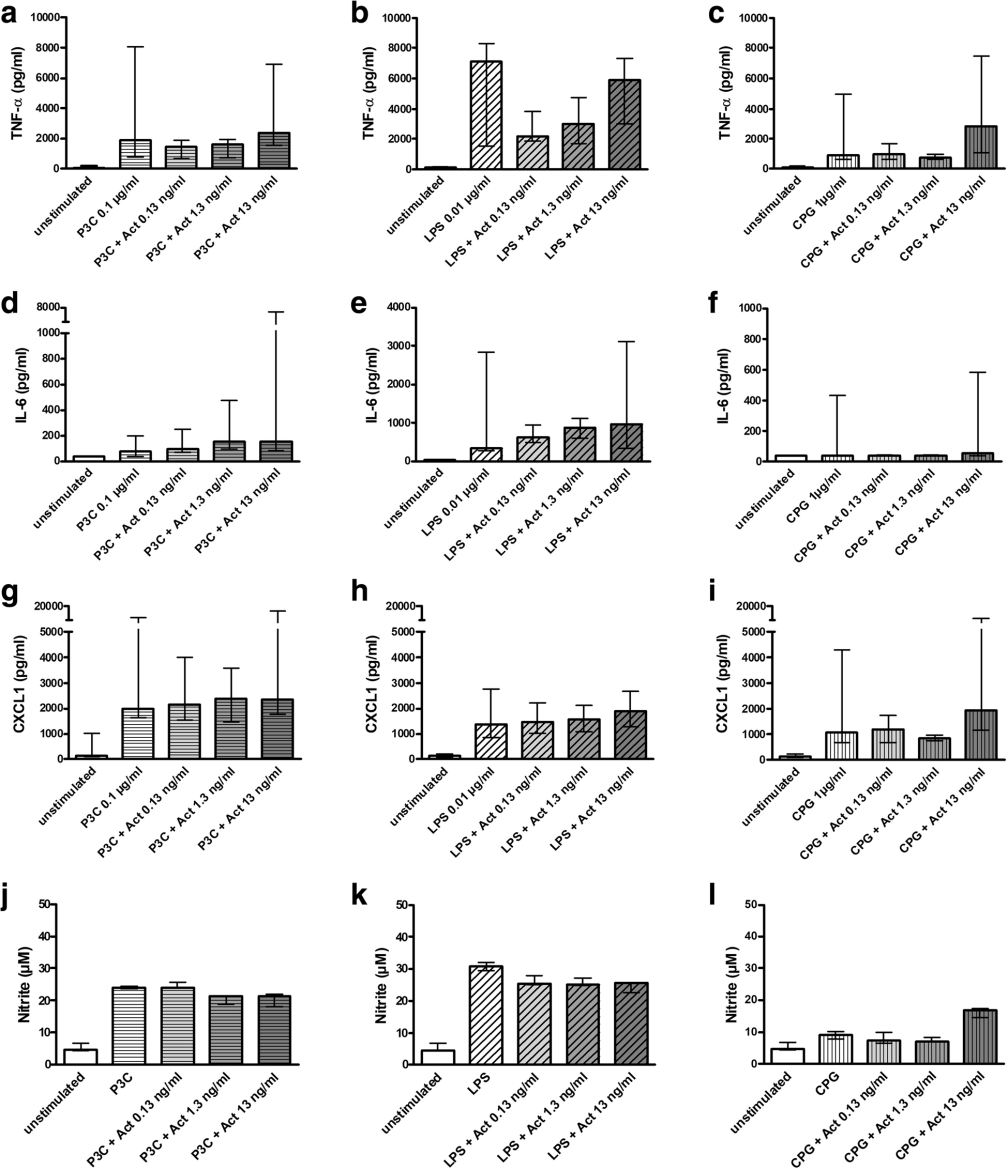
Release of proinflammatory cytokines and NO by microglial cells treated with phagocytosis-enhancing concentrations of activin A and different TLR agonists. Comparison of concentrations of TNF-α (pg/ml) (**a**–**c**), IL-6 (pg/ml) (**d**–**f**), CXCL1 (pg/ml) (**g**–**i**), and NO (μM) (**j**–**l**) in the supernatants of microglial cells after treatment with activin A in concentrations of 0.13, 1.3, and 13 ng/ml in combination with the TLR2 agonist Pam_3_CSK_4_ (P3C; 0.1 μg/ml; **a**, **d**, **g**, **j**), the TLR4 agonist LPS (0.01 μg/ml; **b**, **e**, **h** / 0.003 μg/ml; **k**) and the TLR9 agonist CpG (1 μg/ml; **c**, **f**, **i** / 0.1 μg/ml; **l**) and of microglial cells treated with the respective TLR agonist alone [**a**–**c**: *n* = 8–28 from up to 6 experiments; **d**–**i**: *n* = 8–17 from up to four experiments; **j**–**l**: *n* = 3 from one single experiment]. Data are presented as medians with interquartile ranges and were analyzed by Kruskal-Wallis test followed by Dunn’s multiple comparison test. No significant differences were detected. Please mind the interrupted *y*-axis in **d **and **g**–**i**

As known from previous studies [18–20], the TLR agonists Pam_3_CSK_4_, LPS, and CpG enhanced the release of TNF-α, IL-6, CXCL1, and NO by primary murine microglial cells. Additional treatment of microglial cells with activin A in concentrations of 0.13, 1.3, and 13 ng/ml starting 24 h before stimulation with TLR agonists did not significantly influence the release of TNF-α (Fig. 4a–c), IL-6 (Fig. 4d–f), CXCL1 (Fig. 4g–i), and NO (Fig. 4j–l) compared to treatment with the respective TLR agonists alone.

### In a higher concentration, activin A influenced the release of TNF-α and NO by microglia activated with TLR agonists

Treatment of microglial cells with activin A alone in a high concentration of 13 μg/ml did not significantly influence the release of TNF-α, IL-6, and CXCL1, and NO (Table 1). Additional treatment with 13 μg/ml activin A decreased TNF-α release of LPS-stimulated microglial cells (*p* < 0.0003) and increased NO release of microglial cells stimulated with Pam_3_CSK_4_ (*p* = 0.03) and LPS (*p* = 0.02). No further significant influences of 13 μg/ml activin A on cytokine and NO release were observed (Table 1).

### Activin A did not influence the viability of primary murine microglial cells

**Fig. 5:**
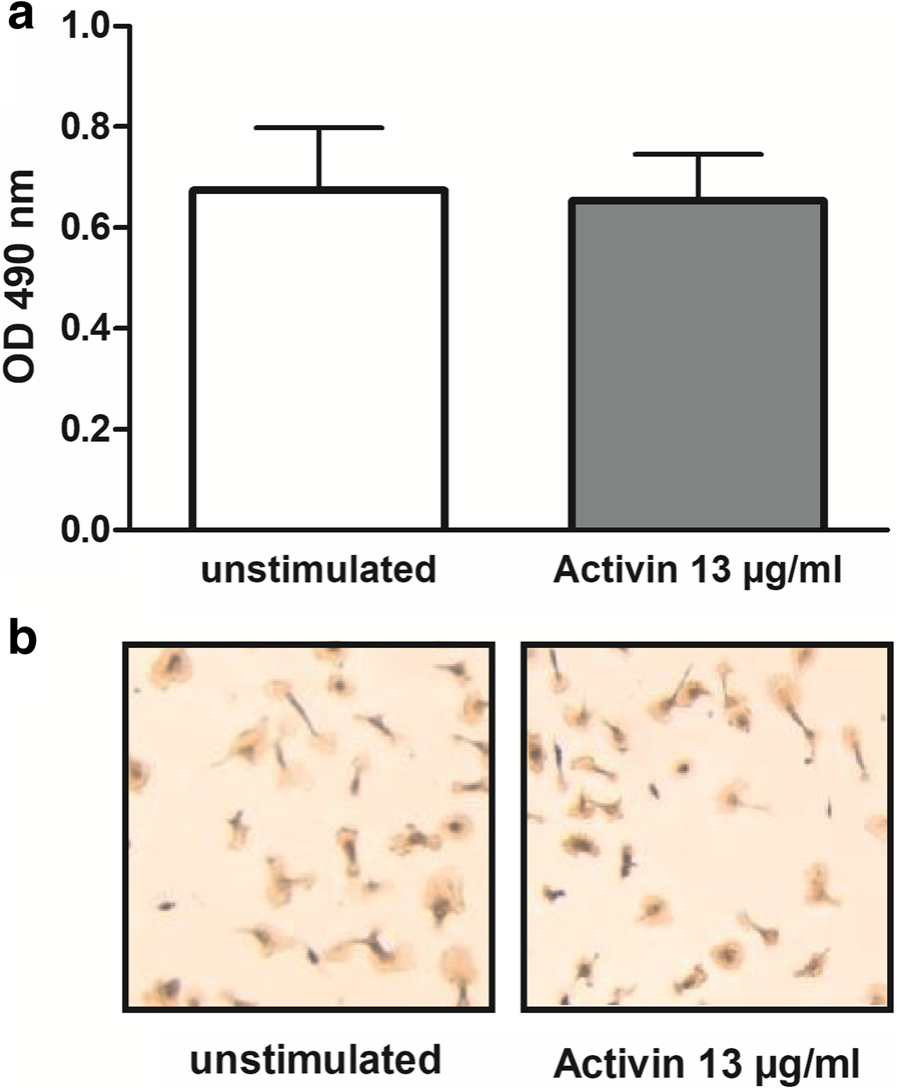
Viability of primary murine microglial cells after treatment with a high concentration of activin A. **a** Comparison of WST-1 assay results of unstimulated primary murine microglial cells and microglial cells after treatment with 13 μg/ml activin A for 48 h (*p* = 0.69). Data are presented as means + SD of the optical density (OD) at 490 nm (*n* = 9 from three experiments). **b** Representative isolectin B4 stainings of unstimulated primary murine microglial cells and microglial cells after treatment with 13 μg/ml activin A for 48 h. By light microscopy, no differences in morphology and density of microglial cells were detected after treatment with activin A

Viability of microglial cells was not influenced by treatment with high doses of activin A (13 μg/ml) as assessed by the WST-1 assay [unstimulated cells (*n* = 9): 100 ± 11%, activin A 13 μg/ml (*n* = 9): 96.9 ± 11.5%, *p* = 0.56; Fig. 5a]. Activin A in lower doses and in combination with the TLR agonists Pam_3_CSK_4_, LPS, and CpG also did not reduce WST-1 values (*n* = 3 per group; *p* > 0.05). Isolectin B4 staining did not reveal changes of morphology or density of microglial cells after treatment with activin A (Fig. 5b).

## Discussion

In search of compounds that increase phagocytosis of pathogens without exerting collateral damage to the brain tissue with the aim to improve the resistance of the brain to infections, we investigated the effect of activin A on primary murine microglial cells. Activin A appears particularly interesting for modulation of brain immune cell functions as neuroprotective effects have been described in vitro [42] and in vivo. Intracerebroventricular (i.c.v.) infusion of activin A in mice had anti-inflammatory and neuroprotective effects in a stroke model [43], enhanced neurogenesis after excitotoxic neurodegeneration [37], and only recently has been shown to protect midbrain neurons in mouse models of Parkinson’s disease (MPTP and LPS model) [44].

We used different concentrations of activin A in our study. The lower concentrations from 0.13 to 13 ng/ml covered the range of concentrations used in the majority of in vitro experiments with different types of immune cells (for review, see [28]) and also the concentrations of activin A measured in human cerebrospinal fluid (CSF) of persons with an age over 60 years without any neurological disease (median 0.14 ng/ml, maximum value 0.33 ng/ml [34]) and mainly younger patients with meningitis (median 0.13 ng/ml [34]) or traumatic brain injury (0.2 ng/ml [45]). To exclude direct toxic effects, we investigated the effect of a 1000-fold higher concentration of activin A (13 μg/ml) which is unphysiolgically high and higher than the estimated CSF concentrations (based on a mouse CSF volume of 35 μl [46]) achieved in mouse experiments by intracerebroventricular (i.c.v.) injection (15 μg/kg bodyweight [43]) or continuous infusion (295 ng/day [44]).

By light microscopic evaluation of isolectin B4 stainings and the WST-1 cell viability assay, we did not detect effects of activin A on viability and morphology of primary microglial cells and could exclude cytotoxic effects of even high activin A concentrations. Similarly, activin A in concentrations from 0.8 to 10 ng/ml did not affect viability and proliferation of cells from a murine macrophage cell line and of primary murine macrophages as assessed by quantitating viable cells with the MTT test which is based on the same principles as the WST-1 test used in our study. Other studies documented an influence of activin A on the density of microglial cells in vitro and in vivo that might be explained by the different experimental settings. In rat microglial cells in vitro, treatment with 10 ng/ml activin A for 24 h enhanced proliferation [36], whereas in primary murine microglial cells, treatment with 100 ng/ml and 1 μg/ml activin A reduced cell numbers alone and in combination with LPS [37]. It also inhibited LPS-induced morphological changes, but did not influence morphology/phenotype of microglial cells when given alone [37]. In mouse models in vivo, i.c.v. administration of activin A alone did not affect numbers of microglial cells; however, continuous administration of activin A for 7 or 15 days after intracerebral challenge with LPS reduced LPS-induced proliferation of microglial cells in specific brain regions [37, 44]. Shorter periods of activin A exposure are more relevant for acute intracerebral infections and might not influence microglial proliferation.

The effect of activin A on phagocytic activity of different phagocytes, mainly macrophages, monocytes [28, 40], and neutrophils [47], has been addressed before; however, to our knowledge, this is the first study investigating the effect of activin A on phagocytic activity of microglial cells. Other groups examined phagocytosis of chicken red blood cells (cRBC), latex particles, and microspheres or pinocytosis of natural red, whereas we investigated phagocytosis of living bacteria. Activin A alone did not significantly affect phagocytosis of *E. coli* K1 by primary murine microglial cells. Differently to these results in resting microglia, in resting murine macrophages (peritoneal macrophages and a macrophage cells line) activin A increased the capacity to phagocytose cRBC and microspheres [38–40]. As in our previous in vitro experiments [18], activation of microglial cells with agonists of TLR 2, 4, and 9 (Pam_3_CSK_4_, LPS, and CpG) increased phagocytosis of *E. coli* K1. Concentrations of TLR agonists which increased the phagocytosis rate approximately 2- to 3-fold were chosen. Treatment with activin A in concentrations of 1.3 and 13 ng/ml starting 24 h before TLR activation, further increased phagocytosis of *E. coli* K1 up to 3.4-fold compared to treatment with the respective TLR agonist alone. Thus, microglial cells treated with activin A in these concentrations followed by activation with one TLR agonist ingested up to 9-fold more bacteria than unstimulated microglial cells. The higher concentration of 13 μg/ml activin A did not influence phagocytosis. In preliminary experiments, we had tested phagocytosis of *E. coli* by microglial cells after treatment with a broader range of activin A concentrations (0.0013 pg/ml to 1.3 μg/ml in intervals of factor ten) in combination with Pam_3_CSK_4_: Bacterial uptake was increased after treatment with Pam_3_CSK_4_ in combination with activin A 1.3 and 13 ng/ml; higher or lower concentrations of activin A neither increased nor decreased bacterial uptake (*n* = 3 per group, data not shown). This shows that the effect of activin A on phagocytic activity is dose dependent. The phagocytosis-inducing effect of activin A differed only slightly between the different TLR agonists and was strongest in case of co-treatment with the TLR2 agonist Pam_3_CSK_4_, followed by CpG and LPS (approximately 2-fold increase compared to LPS alone). In contrast, in mouse macrophages, activin A inhibited the phagocytic activity of LPS-activated macrophages [40, 41, 48]. Reasons for these differences might be the different cells types studied and the different timing of activin A treatment: In our experiment, activin A treatment of microglial cells started 24 h before administration of the different TLR agonists, whereas macrophages in the other studies were simultaneously treated with activin A and LPS. Furthermore, the viable *E. coli* K1 used in our phagocytosis experiments can induce a strong immune reaction itself involving signaling pathways that are not activated by non-bacterial particles used in the other studies. Activin A in combination with the different TLR agonists increased phagocytosis of *E. coli* K1 by primary murine microglial cells up to 9-fold and thus to a similar extent as pretreatment with PEA which subsequently has been shown to reduce bacterial titers and mortality in our mouse model of *E. coli* meningitis [26].

The release of the proinflammatory cytokines TNF-α, IL-6, and CXCL1 from primary murine microglial cells was not increased after treatment with activin A concentrations that enhanced phagocytosis (0.13, 1.3, and 13 ng/ml) or a much higher concentration of activin A (13 μg/ml). Activin A also did not further enhance cytokine release induced by Pam_3_CSK_4_, LPS, and CpG. TNF-α even showed a tendency to be decreased upon activin A treatment. These results are in line with data from previous studies in which activin A downregulated the expression of proinflammatory cytokines in murine primary microglial cells [49] and decreased the LPS-induced release of TNF-α, IL-6, and IL-1 in primary microglial cells of mice and rats [36, 37]. However, in resting mouse macrophages, activin A increased the release of IL-1 and IL-6 [38, 39].

NO production upon microglial activation contributes to microglia-mediated neuronal injury [21–25]. NO release from primary mouse microglial cells was not increased after treatment with activin A alone. Activin A in phagocytosis-enhancing concentrations of 0.13, 1.3, and 13 ng/ml also did not increase NO release induced by Pam_3_CSK_4_, LPS, and CpG. However, in a high concentration of 13 μg/ml, activin A slightly increased NO release induced by Pam_3_CSK_4_ and LPS. In previous studies, activin A has been shown to decrease iNOS mRNA expression in LPS-activated rat microglia [36] and NO release of LPS-activated mouse peritoneal macrophages [41]; however, it enhanced NO release of resting macrophages [38, 39, 50, 51].

Our results indicate that despite multiple similarities of microglial cells and macrophages, there are considerable differences between these cell types concerning the modulation of their functions by activin A. Furthermore, concentration and timing of activin A treatment seem to be crucial parameters for activin A effects. Concentrations of activin A that increased phagocytosis of TLR-activated microglia in vitro are higher than CSF concentrations of activin A that have been measured in patients with meningitis [34]. Thus, endogenous activin A released during bacterial infections probably is not sufficient to influence phagocytic activity of microglial cells. Additional activin A administration is required to achieve an effect on phagocytosis. As described above, i.c.v. administration of activin A has already been shown to be beneficial in mouse models of stroke, excitotoxic neurodegeneration, and Parkinson’s disease [37, 43, 44]. Concentrations that increase NO release under infectious conditions are unlikely to be achieved in vivo.

In mouse macrophages, activin A influenced the expression of TLR, and LPS upregulated the mRNA of Act-RII and Smad 2/3 indicating a mutual regulation of signaling pathways [40]. Timing of activin A administration—before, simultaneously or after an inflammatory stimulus—appears to be critical for activin A effects. In our experimental setting, activin A was administered 24 h before microglial activation by agonists of TLR2, 4, and 9 and 48 h before addition of *E. coli* resembling a preventive approach. TLR2, 4, and 9 are the principal TLR involved in the pathogenesis of bacterial infections which are activated in microglia during the early phase of bacterial CNS infection [10]. Thus, we assessed microglial functions under conditions mimicking a patient at high risk or in an early phase of an already ongoing bacterial CNS infection. In a mouse model of stroke, both prophylactic i.c.v. administration of activin A 1 h before as well as administration 6 h after acute cerebral ischemia had anti-inflammatory and neuroprotective effects [43].

In summary, we demonstrated that activin A dose-dependently enhances phagocytosis of *E. coli* K1 by primary microglial cells activated by different TLR agonists. In phagocytosis-enhancing concentrations, activin A does not increase the release of different proinflammatory cytokines and NO. This suggests that there is no risk of aggravating neuronal injury by the prophylactic or therapeutic administration of activin A. Higher phagocytic activity of microglia without exerting collateral damage to the brain might lead to faster bacterial clearance and prevention or a better outcome of bacterial CNS infections.

## Conclusions

Because of its known neuroprotective effects, activin A appears to be particularly promising for modulation of brain immune cell functions. Our present cell culture results suggest that priming of microglial cells with activin A could increase the elimination of bacteria in bacterial CNS infections. This strategy could improve the resistance of the brain to infections, particularly in elderly and immunocompromised patients. The potential effect of prophylactic activin A administration needs to be investigated in models of bacterial CNS infections in vivo.

## Abbreviations

ABC: Avidin-biotin complex
Act-RI: Activin A receptor type I
Act-RII: Activin A receptor type II
ANOVA: Analysis of variance
BSA: Bovine serum albumin
CFU: Colony-forming units
CNS: Central nervous system
CpG: Cytosine-guanosine motifs
cRBC: Chicken red blood cells
CSF: Cerebrospinal fluid
CXCL1: Chemokine (C-X-C-motif) ligand 1
DMEM: Dulbecco’s modified Eagle’s medium
DNA: Deoxyribonucleic acid
ELISA: Enzyme-linked immunosorbent assay
*E. coli*: *Escherichia coli*
FCS: Fetal calf serum
GRO α: Growth-related oncogene alpha
i.c.v.: Intracerebroventricular
IL-1: Interleukin-1
IL-6: Interleukin-6
iNOS: Inducible nitric oxide synthase
KC: Keratinocyte chemotractant
LPS: Lipopolysaccharide
MPTP: 1-Phenyl-4-methyl-1,2,3,6-tetrahydropyridine
mRNA: Messenger ribonucleic acid
NO: Nitric oxide
OD: Optical density
ODN: Oligodesoxynucleotide
Pam_3_CSK_4_: Tripalmitoyl-S-glycerl-cysteine
PAMPS: Pathogen-associated molecular patterns
PBS: Phosphate-buffered saline
PEA: Palmitoylethanolamide
ROS: Reactive oxygen species
SD: Standard deviation
TGF-β: Transforming growth factor beta
TLR: Toll-like receptor
TNF-α: Tumor necrosis factor alpha
WST: Water-soluble tetrazolium salt
